# Integrative approaches to the prediction of protein functions based on the feature selection

**DOI:** 10.1186/1471-2105-10-455

**Published:** 2009-12-31

**Authors:** Seokha Ko, Hyunju Lee

**Affiliations:** 1 Department of Information and Communications, Gwangju Institute of Science and Technology, Gwangju, Republic of Korea

## Abstract

**Background:**

Protein function prediction has been one of the most important issues in functional genomics. With the current availability of various genomic data sets, many researchers have attempted to develop integration models that combine all available genomic data for protein function prediction. These efforts have resulted in the improvement of prediction quality and the extension of prediction coverage. However, it has also been observed that integrating more data sources does not always increase the prediction quality. Therefore, selecting data sources that highly contribute to the protein function prediction has become an important issue.

**Results:**

We present systematic feature selection methods that assess the contribution of genome-wide data sets to predict protein functions and then investigate the relationship between genomic data sources and protein functions. In this study, we use ten different genomic data sources in *Mus musculus*, including: protein-domains, protein-protein interactions, gene expressions, phenotype ontology, phylogenetic profiles and disease data sources to predict protein functions that are labelled with Gene Ontology (GO) terms. We then apply two approaches to feature selection: exhaustive search feature selection using a kernel based logistic regression (KLR), and a kernel based *L1*-norm regularized logistic regression (KL1LR). In the first approach, we exhaustively measure the contribution of each data set for each function based on its prediction quality. In the second approach, we use the estimated coefficients of features as measures of contribution of data sources. Our results show that the proposed methods improve the prediction quality compared to the full integration of all data sources and other filter-based feature selection methods. We also show that contributing data sources can differ depending on the protein function. Furthermore, we observe that highly contributing data sets can be similar among a group of protein functions that have the same parent in the GO hierarchy.

**Conclusions:**

In contrast to previous integration methods, our approaches not only increase the prediction quality but also gather information about highly contributing data sources for each protein function. This information can help researchers collect relevant data sources for annotating protein functions.

## Background

Due to extensive efforts during the past decades, the genome sequences of many species have been completed. However, researchers have realized the limitations of knowing the genome sequence information if there is no functional information included. For this reason, assigning functions to unknown proteins has become one of the most important topics in functional genomics; although experimental methods have identified a number of protein functions with high accuracy, these approaches have required extensive resources in terms of both time and labour. Therefore, computational methods are necessary to overcome the limitations of these experiments. To this end, computational approaches were first proposed by using the protein sequences, which allowed for the identification of homologous proteins and then to infer specific protein functions [1,2]. Determining the structures of proteins has also identified several interactions between proteins, which subsequently enabled the prediction of protein functions [3]; see a review [4] for the computational methods used for predicting protein functions based on protein structures and protein-protein interactions. In addition, genomic data sets such as protein domain data, protein-protein interaction, gene expression data, phylogenetic profile, phenotype ontology, disease data, and protein complex data have been extensively used to predict protein functions.

Many researchers have also attempted to develop integration models based on various machine learning algorithms and statistical approaches [5-19]. For example, Deng *et al. *[6,7] proposed a Markov random field method for predicting protein function by integrating protein-protein interaction and protein domain data sets. And integrative models based on the kernel were introduced by [8,9] and heterogeneous data sources were integrated in a Bayesian framework for function prediction [10,11]. It should be noted that these methods have outperformed previous approaches that used only one data source [20,21], and that their coverage has also been extended because different data sources can cover the lack of information in others. However, Lu *et al. *and other groups [20,22] showed that the integration of all available data sources is not always the most effective method for increasing prediction quality. For instance, for a given protein function, some data sources highly contribute to improving prediction performance, though others rarely do. Therefore, selecting the most relevant set of data sources is very important in precisely predicting protein function, instead of attempting to use all available data sources.

Feature selection methods have also been applied to many other computational biology problems. For example, to predict protein-protein interactions using protein sequence order information and protein properties, Liu *et al. *[23] selected important features by combining filer-based and wrapper-based feature selection methods. Also, feature selection approaches such as a correlation-based feature subset selection (CFSS) method have been applied to protein structure predictions and protein folding rate predictions to reduce the dimensionality of the protein sequences [24,25]. In any case, all of these studies have demonstrated the importance of feature selection. In this paper, we further posit that the selection of different genomic data sets dependent on the GO term is important for accurate protein function prediction.

The majority of protein function prediction methods have focused on non-mammalian model organisms, and it has not been made clear how accurate function predictions can be made for mammalian organisms. To investigate these problems, nine bioinformatics teams performed an evaluation process using diverse computational approaches in *Mus musculus *to predict protein function based on the integrated data sources [26]. They confirmed that computational approaches integrating various genomic data sets are quite promising for protein function predictions in mammals.

In this study, we investigate the relationship between genomic data sources and protein functions in order to select a group of highly contributed data sources for function prediction in *Mus musculus *and to increase the prediction quality. Here, Gene Ontology (GO) terms are used as the protein function labels and the ten different genomic data sources in *Mus musculus *include protein-domains, protein-protein interactions, gene expressions, phenotype ontology, phylogenetic profiles and diseases. We then apply two feature selection approaches to improve the prediction quality. In the first approach, the contribution of each data source is measured based on its prediction accuracy of the AUC (i.e., the area under a curve of the sensitivity and false positive rate) using a kernel based logistic regression (KLR) method [5]. We repeat the prediction of *1,726 *Gene Ontology Biological Process (GO-BP) terms ten times, where each prediction is performed using a different data source. We then select the data source that provides the highest prediction quality for each GO term; we refer this method as an exhaustive search feature selection approach. As a systematic feature selection method, we next introduce a kernel based *L1*-norm regularized logistic regression (KL1LR) method. In this method, the coefficients of non-contributing features shrink to zero so that we can evaluate the importance of the data source for each protein function. KL1LR outperforms the exhaustive search approach especially for specific GO terms covered by the small number of proteins. We subsequently analyze the contributing data sources obtained using these two approaches. Generally, the protein domain data set is an important data source for a large number of GO functions, though protein-protein interaction and phenotype data sets are also important features of many GO terms. We also investigate whether or not there is agreement between parents and off-springs in the GO hierarchy for most of the contributing data sets. We are able to find many GO terms, in which most off-springs of the given GO term are predicted accurately using the same data set.

Finally, we apply a filter-based feature selection method, Relief [27], and compare the performances between the proposed approaches in this paper and the Relief method. It is found that the proposed methods outperform the Relief method. In addition, consistent results are observed when these methods are applied to the protein function prediction for yeast.

The remainder of this paper is organized as follows. In the Methods section, we first present the descriptions of the various data sources and the definition of kernels for measuring similarities between two proteins. Then, we introduce two different feature selection approaches before describing an enrichment test for detecting the contributing genomic data sets for each GO function. In the Results section, we compare the prediction quality of proposed methods and then, we show the results of grouping GO terms according to the contribution of each data source. Finally, we summarize the contribution of our approach and discuss the limitations of our approach.

## Methods

### Data sources

Throughout this study, ten data sources across six different genomic data types of *Mus musculus *were used: three data sets from gene expressions, two data sets from protein annotations, one protein-protein interaction data set, one phenotype ontology data set, two phylogenetic profile data sets, and one disease association data set. All data sources used were collected during a previous work [26].

#### 1. Gene expression

Three different data sources of gene expression were used and all data sources only contain the data with probes/tags mapping to Mouse Genome Informatics (MGI) genes. The relationship of mapping from probes/tags to MGI genes can be either one to one or many to one. The Zhang *et al. *[28] expression data source includes normalized, median subtracted, and arcsinh intensity values for 13,566 genes across 55 mouse tissues. And the Su *et al. *[29] expression data source includes normalized and gcRMA-condensed values from Affymetrix arrays for 18,208 genes across 61 mouse tissues. In the Zhang *et al. *and Su *et al. *expression data, multiple rows for the same gene are averaged. The SAGE data source contains 99 quality tag counts cut from 139 SAGE libraries for 16,726 genes [30]; in the SAGE data source, we used the average tag count for tags mapped to the same gene.

#### 2. Protein annotations

The Pfam protein-domain [31] and Interpro protein-domain [32] data sets were used. The Pfam data set contains 15,569 genes with 3,133 Pfam-A protein families (release 19), and the Interpro data source contains 16,965 genes with 5,404 sequence patterns (release 12.1) [32]. All these data sources are represented as binary annotation patterns.

#### 3. Protein-protein interactions

The OPHID protein-protein interaction (PPI) data set obtained by orthology (provided by MGI) was used [33]. It contains interactions among 7,125 genes (downloaded in April 2006).

#### 4. Phenotype ontology

The phenotype annotation data set containing 3,439 genes with 33 phenotypes was used [34]. This data source was obtained from MGI and is represented as binary annotation patterns (downloaded in Feb 2006 from [35]).

#### 5. Phylogenetic profile

Conservation patterns indicating whether 15,939 genes have putative orthologues in 18 different species (from yeast to human) were used [36]. These data source were provided by bioMart (v38). The Inparanoid phylogenetic profile data set (v4.0) contains conservation patterns across 21 different species for 15,703 genes [37]; all are represented as binary conservation patterns.

#### 6. Disease associations

Disease association data from the Online Mendelian Inheritance in Man (OMIM) database for *1,938 *genes with *2,488 *diseases were used [38,39]. This data source contains binary annotation patterns (downloaded in Jun 2006 from [40]).

To associate these ten biological data sources with protein functional annotations, Pena-Castillo *et al. *[26] mapped the gene identifiers of each data source to MGI gene identifiers (obtained from MGI in Feb 2006). They used only non-inferred from electronic annotations (IEA) annotations because most IEA annotations are computational predictions that have not been reviewed by curators. From a biological point of view, specific GO terms are interesting, but if GO terms are too specific, there are number of inherent difficulties in making accurate predictions due to the limited number of positive training data sets; therefore, GO-BP terms with {*3-300*} genes annotated in the database were used. In this study, we only used GO terms from a category of biological process, so the final data collection (with GO annotations obtained on Feb 2006) encompassed the information of *21,135 *MGI genes, of which *7,557 *were associated with at least one of the *1,726 *GO terms that we investigated.

In addition, we collected MGI gene identifiers in Aug 2009 and found *2,808 *newly annotated *Mus musculus *genes for 1,051 GO terms since Feb 2006. We predicted these newly annotated genes using the proposed methods to show their prediction quality. Then, to evaluate the performance of our approaches in other model organism, we also used eight genomic data sets and GO terms for yeast obtained from [22]. These data sets consisted of a protein-protein interaction data set, four gene expression data sets, a protein-domain annotation data set, a gene knock-out phenotype data set and a protein localization data set. The GO terms are the Jun 2006 version of the Yeast SGD database; among them, we used 1,246 GO terms covered by {3-300} genes.

### Definition of kernel

We measure the similarity between two genes based on a kernel approach, as we did previously in [5]. In the case of the Pfam and Interpro domain data, we define *v*_*ik *_= 1 if the *i*^*th *^protein has the *k*^*th *^domain, and 0 otherwise. Then, the kernel between the *i*^*th *^and the *j*^*th *^proteins is defined as(1)

where *n *is the number of domains. The kernel is defined similarly for other data sets such as the phenotype ontology, phylogenetic profile, and disease data sets, where a protein is represented by discrete values. On the other hand, kernels for gene expression data sets such as Zhang et al., Su et al., and SAGE gene expression data sources that are represented by continuous values are defined as ***K***^*expression*^(*i*, *j*) = *PCC*(*i*, *j*), the Pearson correlation coefficient between proteins *i *and *j*. Then, the values are discretized to *0 *and *1 *using the threshold *0.3*.

For the protein-protein data set, the following diffusion kernel matrix [5] are used.(2)

where

where *d*_*i *_is the degree of the *i*^*th *^protein in the protein interaction network, *τ *is the diffusion constant, and the diffusion kernel matrix calculates the similarity between all pairs of proteins in the protein interaction network. Here, four different values (*0.1*, *0.5*, *1*, and *3*) were tested as diffusion constants, and *0.1 *was subsequently selected as the optimal diffusion constant (i.e. the diffusion constant *0.1 *gives the best prediction accuracy). Note that in the computed diffusion matrix, values less than the cut-off of *0.001 *were considered as *0*.

### Standardization of data source

Before all the data sources could be integrated, we first standardized each data source since the data sources were each presented in a different scale. The standardized data sources were defined as(3)

where

where *m *is the number of proteins and *n *is the number of features. As a preprocessing step, the standardization results in a zero mean and unit variance for each data feature.

### Kernel-based logistic regression prediction model

In order to integrate ten genomic data sources using feature selection, we propose two approaches based on the kernel-based logistic regression model (KLR) introduced in [5]. In brief, *X*_*i *_= *1 *if the *i*^*th *^protein has the function, otherwise *X*_*i *_= *0*. Let us first explain the case in which a single data set is used. For a given function of interest, a single data set generates two features: 1) the average of similarities between the protein *i *and proteins having the function, and 2) the average of similarities between the protein *i *and proteins not having the function. Then, let the corresponding coefficients *θ *= {*δ*, *ρ*}. Here, the similarity between two proteins *i *and *j *is calculated using the kernel K(i,j). Then, we use the following logistic regression model.(4)

where X_ [-i] _= (*X*_1_, ..., *X*_*i*-1_, *X*_*i*+1_, ..., *X*_*N*_), *N *is the number of proteins, and

This model can be extended for multiple data sets, such that(5)

where *D *is the number of data sets, and  and  can be obtained using Eq. (4) for the *d*-th data set. In this study, we used a glm function (stats package in R) to implement the KRL method.

### Feature selection approaches

In order to investigate factors that highly contribute to protein function prediction and improve the prediction quality, we again introduce two approaches. Both approaches consider one protein function at a time.

#### 1. Exhaustive search

For each data set we estimate the probability of proteins having the function of interest using Eq. (4). We repeat this process for each of ten data sets. In this way, we obtained the accuracy of the predictions for all ten data sets for each GO function.

#### 2. Kernel based L1-norm regularized logistic regression (KL1LR)

The concept of *L1*-norm regularization for feature selection has been widely used in a number of contexts because it relatively easily generates an explainable model for significant features by shrinking some coefficients into zero [41]. For this regularization, let a set of parameters *θ *= {*α*_1_, *β*_1_, ..., *α*_D_, *β*_D_} and rewrite Eq. (5) as follows.(6)

Note that the log likelihood function from the observed data *i *= 1 ... *N*, where *N *is the number of the observed samples, is given as

. By employing the *L1*-norm regularized logistic regression, we can thus estimate the coefficients *θ *by maximizing the log likelihood function and simultaneously penalizing coefficients for features having small contributions to subsequently predict the function.(7)

where a regularization parameter *λ *(>0) controls the cardinality (the number of nonzero components) of *θ*. Generally a larger *λ *tends to yield a smaller cardinality of *θ*, though it also provides a lower prediction accuracy. In an empirical situation, various values of *λ *should be tested to determine the modest trade-off between accuracy and efficiency. In this study, we tested two sets of *λ*, where each set consisted of ten sliding values of *λ*, that were uniformly distributed on a logarithmic scale over the interval [0.1 *λ*_max_, *λ*_max_] and [0.01 *λ*_max_, *λ*_max_]. Here, *λ*_max _was directly calculated from the feature data source. For this study, we used an interior-point method [42] for the implementation of KL1LR since it can be effectively applied to all sizes of problems and has a low time complexity.

### Measurement of prediction performance for each method

To evaluate performance of prediction, we use two-fold cross validation to predict GO terms covered by {*3-10*} genes and five-fold cross validation for GO terms covered by {*10-300*} genes. For a given GO term, the contribution of each feature is measured using training sets and the performance of the test set is then measured by predicting the function based on the selected features. More specifically, for the exhaustive search feature selection approach, the AUC value of each data set is measured using the training set, and the data set with the highest AUC value is then selected as the feature for further testing. For the KL1LR approach, the coefficients of the features are first measured by the training set and the features with non-zero coefficients are selected as the features for testing. Finally, the AUC and precision at 20% recall (P20R) values are calculated for the test set.

### Measurement of contributions of each data source

We measured the contribution of data sources for each GO term and analyzed them. In the exhaustive search approach, data sources providing the high AUC value (≥*0.75*) and the high P20R value (≥*0.2*) for the test set were selected as the highly contributing factors for each GO-BP term. In the case of KL1LR, when the P20R value was greater than or equal to *0.2*, we considered features with regression coefficients located outside the *1 *standard deviation (SD) as the highly contributing factors for protein function prediction.

Next, the enrichment test was employed to obtain a general view of the GO terms that were well predicted by the specific data source. Because GO is a directed acyclic graph, we could derive the properties of specific groups of GO terms by backtracking their common ancestor. In this way, we investigated whether there is an agreement between parents and off-springs in the GO hierarchy for most contributing data sets. In order to test the statistical significance, we performed a hypergeometric test to compare the actual number of descendent GO terms that were well predicted using a given data source with the total number of descendent GO terms.

For this task, we denoted the group *G*_*d *_as the GO terms having high predictive power based on data source *d*. For each GO term in the group *G*_*d*_, let *m, n, k*, and *x *be the number of descendent GO terms, the number of non-descendent GO terms, the number of GO terms in the group *G*_*d*_, and the number of descendent GO terms in the group *G*_*d*_, respectively. Then, the p-value is given by(8)

Through this statistical approach, we could measure how much the contributing data sources of each GO term agreed with those of its descendent GO terms, thereby allowing recognition of the overall tendencies of the GO terms.

## Results

### Performance evaluation

**Figure 1:**
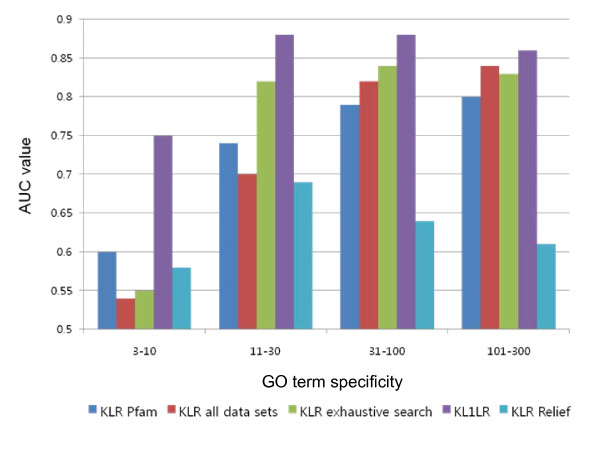
**The AUC values of function predictions based on different prediction approaches**. The AUC values of KLR using Pfam, KLR using all data sets (standardized), KLR using a data source selected by exhaustive search, KL1LR (*λ *= *0.01*, standardized), and KLR using data sources selected by the Relief method are shown.

We first show the significance of feature selection in integrating multiple data sources. Table 1 and Figure 1 show the averages of AUC values of four different categories of GO terms predicted by the KLR method with Pfam or Interpro data only, the KLR method with all data sources, the KLR method with a data source selected by exhaustive search, and the KL1LR method with all data sources. When integrating the data sets, the AUC values for both standardized and non-standardized data are used. In KL1LR, it can be seen that the standardization of integrated data has improved the prediction quality and that the prediction accuracy of the AUC value is higher when the relative parameter *λ *= *0.01 *than when *λ *= *0.1*. On the other hand, performances in the KLR method integrating all data sources with standardization and non-standardization are similar. Hence, we used the KL1LR results with standardization and *λ *= *0.01 *for the study in this paper.

**Table 1:** Prediction quality of different prediction approaches

Specificity	# of GO terms	KLR					KL1LR				KLR with Relief
		Pfam	Interpro	All		ES	*λ*/*λ*_max _= 0.01		*λ*/*λ*_max _= 0.1		
							
				NOS	S		NOS	S	NOS	S	
3-10	952	0.60	0.58	0.55	0.54	0.55	0.73	0.75	0.72	0.74	0.58
11-30	435	0.74	0.76	0.70	0.70	0.82	0.85	0.88	0.79	0.85	0.69
31-100	239	0.79	0.79	0.82	0.82	0.84	0.84	0.88	0.78	0.86	0.64
101-300	100	0.80	0.80	0.84	0.84	0.83	0.82	0.86	0.73	0.84	0.61

GO terms are categorized into four groups based on the number of genes covering the GO term (specificity in the first column). Prediction quality is estimated using AUC values for KLR using Pfam or Interpro data only, KLR using all data sources, KLR using a data source selected by exhaustive search (ES), KL1LR, and KLR using data sources selected by the Relief method. In the case of KL1LR, two different values of the regularization parameter *λ *are used. NOS stands for non-standardization of features, and S for standardization.

Based on the prediction of protein functions using each data set, we found that the domain data sets, including Interpro and Pfam, were the most informative among the six different types of data sets (see Additional File 1 for more information). Surprisingly, the AUC values for the Interpro data set for the categories of {3-10} and {11-30} were higher than the AUC values when all the data sets were integrated. This result is due to the fact that some data sets actually act noise during modelling, thereby confirming the importance of accurate feature selection. To this end, prediction accuracy could be increased here by using the two feature selection approaches of exhaustive search feature selection and KL1LR. The average AUC values of exhaustive search were 0.55, 0.82, 0.84, and 0.83 for the four GO term categories. However, KL1LR significantly outperformed the exhaustive search feature selection for the {3-10} category (0.75) though it had comparable prediction accuracies for the other general categories (0.88, 0.88, and 0.86). As expected, the prediction accuracy was generally lower in the category {3-10} due to the small number of positives compared to negatives. Note that the AUC value of all the GO terms for KLR with each data source, KLR with all data sources, and KL1LR are available in Additional File 1. Precision at various recall values for KLR with each data source and KL1LR (*λ *= *0.01*, standardized data) are available in Additional File 2.

Both exhaustive search feature selection and KL1LR methods are wrapper-based feature selection methods. Therefore, to compare the performances between different feature selection approaches in the genomic data sets, we applied a filter-based feature selection method, Relief [27]. As a pre-processor, Relief is usually used to remove irrelevant attributes from data sets before learning. In Relief, the weight of each feature is calculated by estimating the number of instances a feature can distinguish in the same class from instances in a different class. The features with the shortest distance in the same class and the longest distance in a different class obtain a high weight. Usually, normalized weights are used, at an interval of [-1, 1]. This procedure is then repeated for *n *times, which is a user parameter for determining the number of training instances. In our experiment, the sampling rate was set as 25. We then calculated the weights for all 20 features obtained from kernels using the 10 data sets. Finally, we selected important features displaying more than 0.05 as a weight. These sampling rates and thresholds were determined through experimentation.

After selecting features using the Relief method, we applied the KLR method for further classification. The AUC values of four categories were 0.58, 0.69, 0.64, and 0.61, which show low prediction accuracy (Table 1 and Figure 1). Because mouse function data set contains a small number of positive instances compared to negative instances, this structure-based feature selection technique seems unable to achieve a high prediction accuracy; prediction accuracies were similar although the sampling rates were set to be higher than 25. For all *1,726 *GO terms, prediction accuracies of the AUC values and Relief weights of 20 features are available in Additional File 3.

### Contributions of each data source for protein function predictions

Using the feature selection methods, we improved the prediction performance compared to the method integrating all the data sets. In addition, we were able to determine which data sources are informative for predicting each GO term; further details for each GO term of which are presented in Additional File 2. Here, we investigated how many GO terms are highly accurately predicted using each data source in order to summarize the importance of genomic data sets in protein function predictions.

During this investigation, we selected data sources with high prediction accuracies for each GO term. For the KLR method with exhaustive search, we selected a data set having thresholds of AUC of ≥ 0.75 and P20R of ≥ 0.2 from the test set (see Additional File 2 for the data). For the KL1LR method, we selected data sources having a high regression coefficient (outside of *1 *SD) and a high P20R value (≥ 0.2). These criteria were adjusted so that there were a similar total number of GO terms between the exhaustive search feature selection method and the KL1LR method. Table 2 shows the number of GO terms selected for each data source and the average of the prediction accuracies with the respective data source (see Additional File 4 for the lists of GO terms selected for each data source). In this analysis, we observed that the domain data sources are informative for the largest number of protein functions.

**Table 2:** Contributions of genomic data sources

Data source		Exhaustive search			KL1LR			
		# of GO terms (# in union)		AUC	# of GO terms (# in union)		AUC	NCG
Protein-protein interactions	OPHID	192		0.82	201		0.89	83

Protein domain	Interpro	522	(697)	0.87	408	(518)	0.89	266
	Pfam	600		0.86	311		0.89	210

Phenotype	MGI	213		0.87	346		0.90	129

Phylogenetic profile	BioMart	33	(95)	0.83	59	(166)	0.88	4
	Inparanoid	70		0.84	124		0.88	22

Disease	OMIM	41		0.85	32		0.88	3

Gene expression	Zhang *et al.*	28		0.81	147		0.90	10
	Su *et al.*	21	(55)	0.82	158	(309)	0.89	8
	Sage *et al.*	16		0.83	113		0.90	7

The numbers of GO terms satisfying the cut-off of prediction accuracies by the AUC and P20R values are presented for each data source along with the average AUC values of the GO terms. For the protein domain, phylogenetic profile, and gene expression data, the number of terms in the union set is shown in parentheses. The numbers of common terms between the two approaches are shown in the last column (NCG).

Specifically, using the exhaustive search, 40% of the GO terms (*697 *of *1,726 *terms) have a high prediction accuracy with domain information; this is also the case for the KL1LR method. Also, the protein interaction and the phenotype data sets turned out to be the informative data sources for many of the GO terms. Conversely, phylogenetic profile, disease, and gene expression data sets were only informative for a small fraction of the GO terms; 5% (95/1,726), 2% (41/1,726), and 3% (55/1,726), respectively.

For the exhaustive search feature selection approach, the informative data sources were independently selected from other data sources. However, the KL1LR method tends to shrink the coefficients of similar features to select more relevant features when several redundant data sources are available. Hence, the contribution of each data set might not be accurately detected. The last column in Table 2 indicates the number of GO terms common in two feature selection approaches. As can be seen, the fractions of intersections are pretty high when considering the fact that the features selected by the KL1LR method are affected by the redundant data sources. Among the GO terms selected by using the exhaustive search approach, 51% (266/522), 43% (83/192), and 61% (129/213) of the terms were also selected using the KL1LR approach from Interpro domain data, protein-protein interaction data, and phenotype data, respectively. These observations confirmed that the general importance of genomic data types was consistently observed throughout the two feature selection approaches.

To enhance the understanding of the relationship between protein functions and genomic data sets, we also investigated the group of GO terms that have a high prediction quality based on only one data set. For this task, we selected a set of GO terms using the exhaustive search for each data type, in which the given data source had the highest AUC value for a given GO term; the AUC score was ≥ 0.75 and the P20R value was ≥ 0.2, and the AUC value difference with the second highest AUC value from other data sources was larger than 0.1. Note that if the data sets with the highest and the second highest accuracy were in the same class of data type, we compared the highest prediction accuracy with the third highest accuracy. Here, two protein domain data sets, three gene expression data sets, and two phylogenetic profile data sets are considered as the same type of data. Table 3 shows a part of the results with the entire table being available on Additional File 5. Additional File 6 then presents the data values for the most contributing data set for each GO term. In the following section, we explain examples of GO terms with their respective most contributing data types.

**Table 3:** GO terms giving a high prediction quality using only one data source

Data source	GO	Term	NPWGD	NPWG	AUC	P20R	DA
OPHID (PPI)	0048489	synaptic vesicle transport	13	14	0.92	0.39	0.17
	0006887	exocytosis	21	27	0.91	0.87	0.12

Interpro (Domain)	0006071	glycerol metabolism	10	11	0.87	0.8	0.44
	0000160	two-component signal transduction system (phosphorelay)	10	10	1	1	0.41
	0006801	superoxide metabolism modification-dependent	10	10	0.93	1	0.31
	0043632	macromolecule catabolism	47	47	0.96	0.5	0.28
	0006508	proteolysis	233	240	0.92	0.69	0.28
	0006812	cation transport	173	176	0.90	0.93	0.15

Pfam (Domain)	0016311	dephosphorylation	48	51	0.97	0.81	0.35
	0006338	chromatin remodeling	21	22	0.91	0.28	0.33
	0031497	chromatin assembly protein amino acid	29	30	0.97	0.63	0.32
	0006470	Dephosphorylation	46	49	0.98	0.69	0.31
	0006333	chromatin assembly or disassembly	41	42	0.96	0.71	0.3

MGI (Phenotype)	0008344	adult locomotory behavior	14	19	0.9	0.21	0.31
	0030534	adult behaviour	18	23	0.9	0.21	0.3
	0007605	sensory perception of sound	26	40	0.94	0.55	0.27
	0048232	male gamete generation	44	70	0.93	0.34	0.26
	0007283	spermatogenesis	44	70	0.94	0.28	0.25
	0000003	reproduction	101	152	0.87	0.52	0.20

OMIM (Diseases)	0008643	carbohydrate transport	11	30	0.94	0.87	0.15

Zhang *et al. *(Gene expression)	0001502	cartilage condensation	10	10	0.85	0.23	0.15

GO terms and data sources displaying the outstanding contributions to the prediction of the given GO term are listed, where only part of the lists among the GO terms covering greater than or equal to 10 proteins are presented. NPWGD stands for number of proteins having the given GO term and the given data source, NPWG is the number of proteins with the given GO term, and DA is the difference between the AUC score and the second highest accuracy.

#### 1) Domain

**Figure 2:**
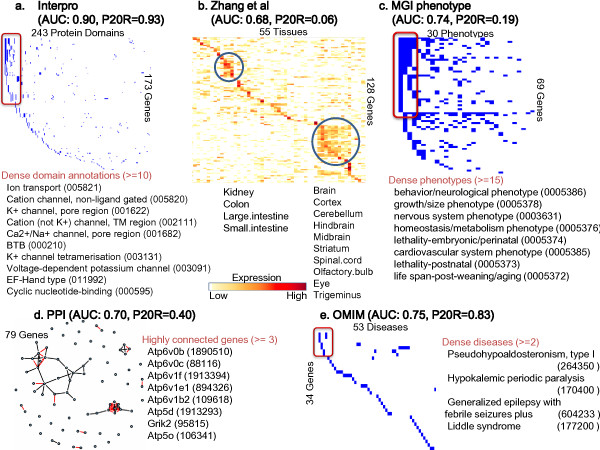
**Illustration of genomic data sources for the genes with 'Cation transport' function**. 176 genes have the 'Cation transport' (GO:0006812) function. The relationship between these genes and the five different genomic data sources are illustrated. For each data source, the AUC value and the P20R value with the KLR method are also represented. (a) Protein domains belonging to the genes with the given GO term are coloured blue in the matrix. Domains appearing in the more than 10 genes are boxed in red in the matrix, and their names and identifiers are listed below. (b) Expression levels of genes with the given GO terms are presented. Genes and tissues are grouped based on the hierarchical bi-clustering of expression levels. Tissues commonly over-expressed in several genes are circled in blue and the names of tissues are listed below. (c) MGI phenotypes belonging to the genes with the given GO term are coloured blue in the matrix. Phenotypes appearing in the more than 15 genes are boxed in red in the matrix, and their names and identifiers are listed below. (d) Protein-protein interaction network of genes with the given GO term. Red (black) lines indicate the direct (indirect) interactions. Highly connected proteins based on direct interactions are listed with their identifiers. (e) OMIM disease is similarly presented its domains and phenotypes.

Interpro is the most informative data set for predicting proteins involved in 'Cation transport' (GO:0006812) as shown in Figure 2. Many genes with this function contain the domains related to ion transport and cation channels such as the K+ channel, which are the hallmark detecting genes for 'Cation transport', which is supported by the Interpro providing the manual assignments of GO terms to each Interpro domain [32].

**Figure 3:**
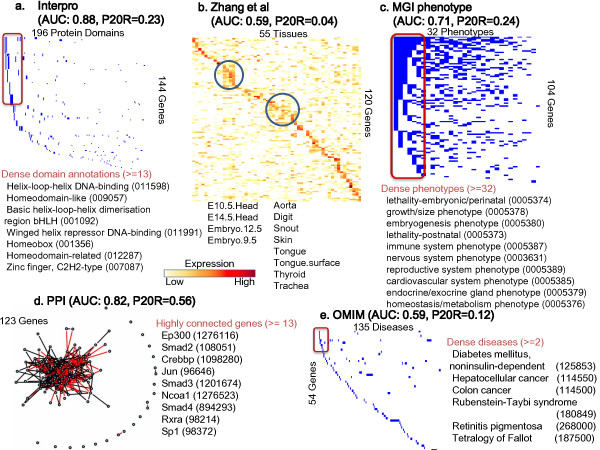
**Illustration of genomic data sources for the genes having a 'Positive regulation of nucleobase, nucleoside, nucleotide and nucleic acid metabolism'**. *161 *genes have the 'positive regulation of nucleobase, nucleoside, nucleotide and nucleic acid metabolism' (GO:0045935) function. (a) - (e) as described in Figure 2 (a) - (e).

The prediction power for the 'Cation transport' using the Interpro protein-domain information is considerably higher than using the other data sets. However, the prediction quality for the 'Positive regulation of nucleobase, nucleoside, nucleotide and nucleic acid metabolism (GO:0045935)' in Figure 3 is higher using both the protein-protein interaction and the Interpro domain data sets. Many genes in this GO category commonly contain domains related to DNA binding such as helix-loop-helix DNA binding (IPR011598, 001092), winged helix repressor DNA-binding (IPR011991), homeobox (IPR009057, 001356, 012287) and zinc finger C2H2 (IPR007087), which might increase the prediction quality using the Interpro data set. Concurrently, genes such as Sp1, Ep300, Smad2, Ncoa1, Rxra, and Crebbp in this category belong to transcription factor complexes, transcriptional regulation, or signal transduction so that they work together with other proteins in the same GO process by physically interacting with each other [43-45], which might increase the prediction quality using the protein-protein data set.

#### 2)Phenotype

**Figure 4:**
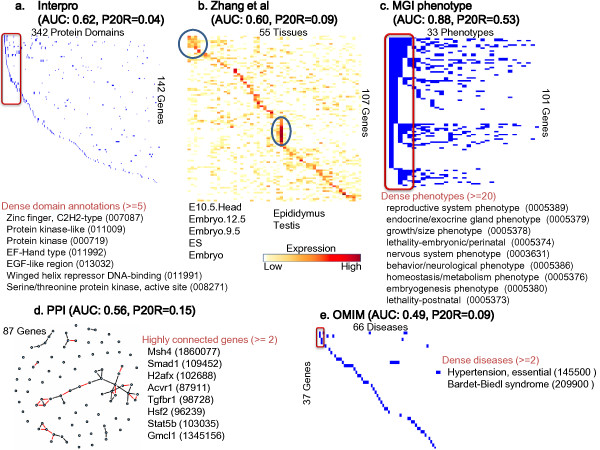
**Illustration of genomic data sources for the genes having a 'Reproduction' function**. *152 *genes have the 'Reproduction' (GO: 0000003) function. (a) - (e) as described in Figure 2 (a) - (e).

Table 3 and Figure 4 show that the MGI phenotype is the most relevant data source for predicting proteins with 'Reproduction (GO: 0000003)'. Most of genes in this category belong to the 'Reproductive system phenotype (0005389)' which is the most powerful indicator for predicting this category. Also, more than 20 genes belong to the same phenotype in 9 phenotype categories, which helps to predict their GO functions. Note that genes in this GO term rarely interact with each other and only a few genes have the same domain. (Additional Files 7, 8, and 9 contain the data sets for Figures 2, 3, and 4.)

#### 3) Gene expression

The prediction accuracy of cartilage condensation (GO:0001502) using Zhang *et al. *[28] gene expression data is very high, with an AUC value of 0.85, a P20R value of 0.225, and the difference from the second highest AUC value is 0.15. Genes with cartilage condensation function are commonly highly expressed in the tissues of E14.5. Head, femur, snout, teeth, thyroid, and trachea (see Additional File 6), most of which are tissues related to cartilage. Cartilage is a tissue found in the organs such as femur, snout, and thyroid gland, and trachea is held open by15~20 C-shaped rings of cartilage [46]. Genes belonging to this GO term such as CTGF, Col11a1, Ror2, and Thra are known to be expressed in skeletal cells [47], are essential genes for skeletal morphogenesis and skeletal system development [48,49], and are related to bone formation [50].

### An enrichment test for an informative data source in a GO hierarchy

It could be observed in Table 3 and Additional File 6 that the GO terms highly accurately predicted with one data source are sometimes in a parent-offspring relationship. To obtain a general view of the relationship between GO terms and genomic data sources, we performed an enrichment test on an informative data source in the GO hierarchy, where the majority of the offspring GO terms of a given GO term were highly accurately predicted using the same data type. For this task, we used hypergeometic test described in the Method section. Table 4 summarizes parts of the results based on the exhaustive search feature selection approach. Because the informative data sources for each GO term might differ between the exhaustive search feature selection method and the KL1LR method, the significant GO terms in the enrichment test detected by the KL1LR method are highlighted in bold and italic type. It should be noted that for some GO terms, the data source was informative for the majority of the GO term's off-spring, but not informative for the given GO term itself. In this case, the informative data source for the GO term itself from both approaches is underlined. The entire table is available in Additional File 10.

**Table 4:** Enrichment test for an informative data source in the GO hierarchy

Data source	GO term	Description	P-value	NO	NOPW
OPHID (PPI)	***0001775***	***cell activation***	***1.24E-08***	***69***	***24***
	***0046649***	***lymphocyte activation***	***1.47E-07***	***57***	***20***
	***0016070***	***RNA metabolic process***	***1.90E-07***	***44***	***17***
	***0045321***	***leukocyte activation***	***3.03E-07***	***64***	***21***
	***0046651***	***lymphocyte proliferation***	***8.95E-07***	***16***	***9***

Interpro (Domain)	***0006811***	***ion transport***	***2.68E-07***	***23***	***18***
	***0006812***	***cation transport***	***5.32E-07***	***15***	***13***
	***0006807***	***nitrogen compound metabolic process***	***7.76E-07***	***57***	***34***

Pfam (Domain)	0044271	nitrogen compound biosynthetic process	0	10	10
	***0006807***	***nitrogen compound metabolic process***	***3.98E-11***	***57***	***43***
	***0009308***	***amine metabolic process***	***2.47E-09***	***52***	***38***
	***0006519***	***amino acid and derivative metabolic process***	***5.53E-09***	***51***	***37***
	0006725	aromatic compound metabolic process	4.85E-08	25	21
	***0006520***	***amino acid metabolic process***	***5.36E-08***	***30***	***24***
	***0006091***	***generation of precursor metabolites and energy***	***1.25E-07***	***15***	***14***
	***0006811***	***ion transport***	***3.21E-07***	***23***	***19***

MGI (Phenotype)	***0001775***	***cell activation***	***3.42E-08***	***69***	***25***
	***0046649***	***lymphocyte activation***	***2.61E-07***	***57***	***21***
	***0045321***	***leukocyte activation***	***6.25E-07***	***64***	***22***

BioMart (Phylogenetic profile)	0046164	alcohol catabolic process	1.21E-08	6	4
	0030100	regulation of endocytosis	5.89E-07	5	3
	0046365	monosaccharide catabolic process	5.89E-07	5	3

Inparanoid (Phylogenetic profile)	***0001775***	***cell activation***	***3.45E-11***	***69***	***17***
	***0051239***	***regulation of multicellular organismal process***	***3.58E-11***	***88***	***19***
	***0050865***	***regulation of cell activation***	***2.70E-09***	***42***	***12***
	***0046649***	***lymphocyte activation***	***1.76E-08***	***57***	***13***
	***0045321***	***leukocyte activation***	***8.80E-08***	***64***	***13***
	***0019220***	***regulation of phosphate metabolic process***	***1.93E-07***	***13***	***6***
	***0051174***	***regulation of phosphorus metabolic process***	***3.74E-07***	***14***	***6***
	***0042325***	***regulation of phosphorylation***	***6.77E-07***	***10***	***5***
	***0051249***	***regulation of lymphocyte activation***	***9.39E-07***	***37***	***9***

OMIM (Diseases)	0006812	cation transport	1.62E-05	15	4
	***0006118***	***electron transport***	***5.17E-05***	***4***	***2***

Zhang *et al *(Gene expression)	0040013	negative regulation of locomotion	3.21E-06	3	2
	***0000279***	***M phase***	***5.70E-06***	***19***	***4***
	***0019882***	***antigen processing and presentation***	***3.14E-05***	***5***	***2***
	0008380	RNA splicing	6.22E-05	6	2
	***0007049***	***cell cycle***	***8.95E-05***	***52***	***5***

Su *et al *(Gene expression)	***0050953***	***sensory perception of light stimulus***	***5.59E-06***	***4***	***2***
	***0019882***	***antigen processing and presentation***	***1.39E-05***	***5***	***2***
	0007059	chromosome segregation	1.39E-05	5	2

SAGE (Gene expression)	***0019882***	***antigen processing and presentation***	***4.45E-06***	***5***	***2***
	0050851	antigen receptor-mediated signaling pathway	1.54E-05	7	2
	***0042110***	***T cell activation***	***3.93E-05***	***26***	***3***
	***0030217***	***T cell differentiation***	***5.21E-05***	***10***	***2***
	***0019395***	***fatty acid oxidation***	***6.34E-05***	***2***	***1***

The data source in the first column is informative for predicting gene functions belonging to the GO terms in the second column and its off-spring GO terms. The p-values in the fourth column represent the significance of the number of off-springs that are well predicted using the given data source. This table presents GO terms having p-value <*1.00E-04 *and having a sufficient number of off-springs. The bold and italic fonts indicate the significant GO terms based on the enrichment tests from the exhaustive search feature selection and the KL1LR method for the given data source. Among them, if the data source is informative for the GO term itself, the GO term is underlined (see the main text for more explanation). NO stands for the number of all off-springs of a GO term and NOPW for the number of off-springs predicted well using a given data source.

**Figure 5:**
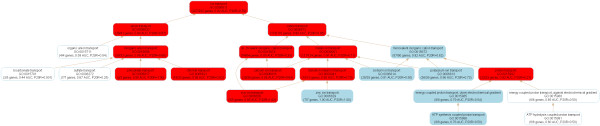
**The hierarchy of 'Ion transport' (GO:0006811)**. Coloured GO terms have high AUC values based on the Interpro domain data set. Among them, the red boxes represent GO terms having a high AUC in only that data source. In parentheses, the number of genes having the given GO term (the number of genes having the Interpro data source is also represented), the AUC values, and P20R values having the Interpro data source are represented.

From Table 4, we observed that the informative data sources for the GO terms were not independent of each other. For example, 'Ion transport' (GO:0006811, *232 *genes) in Figure 5 is a highly significant term in the enrichment test with the Interpro domain data set. Among the 23 off-spring GO terms of "Ion transport", 18 off-springs have high prediction accuracy when the Interpro data set is used, giving a p-value of 0.000000268. 'Cation transport' (GO:0006812, *176 *genes) in Figure 2, which has a high AUC value when using only the Interpro data set, is one of the off-spring of 'Ion transport'. Similarly, 'Reproduction' (GO:0000003, *152 *genes) was found to be a significant term in the MGI phenotype; the GO term hierarchy of 'Reproduction' (GO:0000003) is also available in Additional File 11. In total, 17 out of 47 descendent GO terms could be well predicted using the MGI phenotype.

### Performance of our prediction approach for newly annotated genes in *Mus musculus *and yeast

As one of the performance evaluation approaches for our feature selection methods, we collected MGI gene identifiers on Aug 2009 and predicted *2,808 *genes newly annotated since Feb 2006. We tested these genes using both KLR with exhaustive search feature selection and KL1LR methods based on selected features and the trained parameters using data sets collected on Feb 2006. As a result, it was found that among the *1,726 *GO terms, *1,051 *had newly annotated genes.

Table 5 shows the prediction results for the newly annotated genes. In these results, the prediction accuracies of newly annotated genes using the KL1LR method are quite high; AUC values are *0.79*, *0.81*, *0.82*, and *0.80 *for four categories, respectively. Surprisingly, the AUC values in category {3-10} are also high and are comparable to other categories. Since some inaccurate annotations are discarded from older annotation data, the frequency of false positive and false negative could be decreased, which confirms the robustness of our approaches. The full prediction results and precision at various recall values, including the number of newly annotated genes of each GO term, are available on Additional Files 12 and 13.

**Table 5:** Prediction quality of newly annotated genes

Specificity	# of genes	KLR	L1LR	
		Exhaustive search	*λ*/*λ*_max _= 0.01	
			
			NOS*	S*
3-10	540	0.61	0.76	0.79
11-30	273	0.71	0.79	0.81
31-100	163	0.72	0.80	0.82
101-300	75	0.69	0.78	0.80

GO terms are categorized into four groups based on the number of genes covering the GO term. Prediction quality is estimated by using the AUC values for KLR with a data source selected by exhaustive search and KL1LR with all data sources. In the case of KL1LR, two different values of regularization parameter *λ *are used. NOS stands for non-standardization, and S for standardization.

To evaluate the performance of our approaches for other model organisms, eight genomic data sets from yeast were integrated using the proposed feature selection methods. The data sets consist of a protein-protein interaction data set, four gene expression data sets, a protein-domain annotation data set, a gene knock-out phenotype data set, and a protein localization data set [22]. Table 6 shows the average AUC values of GO terms for four different GO term categories predicted using the KLR with all data sets, KLR with a data source selected by exhaustive search, the KL1LR method, and the Relief method. After the different data sets were integrated, both the standardized and non-standardized data were compared. Compared to prediction results in Table 1 for *Mus musculus *data, the AUC values decreased. However, the integration approach based on the features selected using the exhaustive search and the KL1LR method consistently outperformed the other approaches, such as KLR with all genomic data sets and the Relief feature selection method. From these results, we can confirm that the proposed approaches are also suitable for use with other organisms. The full set of prediction results and precision at various recall values of each GO term are available in Additional Files 14 and 15.

**Table 6:** Performance of GO term prediction of yeast genes

Specificity	# of GO terms	KLR			K L1LR				KLR with Relief
		All		ES	*λ*/*λ*_max _= 0.01		*λ*/*λ*_max _= 0.1		
					
		NOS*	S*		NOS*	S*	NOS*	S*	
3-10	567	0.51	0.51	0.61	0.68	0.70	0.67	0.68	0.52
11-30	348	0.70	0.70	0.79	0.80	0.82	0.78	0.79	0.66
31-100	210	0.75	0.75	0.80	0.79	0.82	0.77	0.79	0.64
101-300	121	0.77	0.77	0.79	0.77	0.80	0.73	0.78	0.65

GO terms are categorized into four groups based on the number of genes covering the GO term. Prediction quality is estimated using AUC values for KLR with all data sources, KLR with a data source selected by exhaustive search (ES), KL1LR method (with two regularization parameters), and KLR with features selected by the Relief method. NOS stands for non-standardization of the features, S for standardization.

### Consistency of informative features in newly annotated genes in *Mus musculus *and yeast genes

We then investigated whether or not the informative genomic data types collected on Feb 2006 were also informative for newly annotated genes. Among the *1,051 *GO terms having newly annotated genes, we selected informative features (AUC ≥0.75) for the newly annotated genes using the exhaustive search feature selection and compared them with those from the Feb 2006 data sets (Addition File 2). Table 7 shows that *506 *GO terms have the same informative feature types for both data sets; full information for Table 7 is available in Additional File 16.

**Table 7:** Consistencies of informative genomic data types between old and new annotation data in *Mus musculus *and between *Mus musculus *and yeast

	*Mus musculus*		Yeast	
Specificity	# of GO terms	# of GO terms having the same informative feature types	# of GO terms	# of GO terms having the same informative feature types
3-10	540	203	270	61
11-30	273	177	176	59
31-100	163	91	119	41
101-300	75	35	57	25
Total	1051	506	622	186

GO terms are categorized into four groups based on the number of genes covering the GO term. The number of genes and the number of GO terms with the same informative feature types with *Mus musculus *Feb 2006 data are presented for newly annotated genes on Aug 2009 for *Mus musculus *and yeast data sets, respectively.

To investigate the consistency of informative genomic data sets in yeast, among the *1,246 *GO terms, we analyzed *622 *GO terms that intersected with *Mus musculus*. Here, the informative features were defined as data sets having ≥ 0.75 AUC. For the analysis, the same types of genomic data sets between *Mus musculus *and yeast were considered; i.e., a protein-domain annotation data set, gene expression data sets, a protein-protein interaction data set, and a phenotype data set. Table 7 shows that *186 *GO terms have the same informative feature types for both data sets; full information including the gene counts in the yeast data for Table 7 is available in Additional File 17.

## Discussion

The contribution of this paper compared to the study by Pena-castillo *et al. *(2006) [26] is that the methods proposed in this paper could systematically select important genomic data types for function prediction and thereby improve the prediction quality based on feature selection. Pena-castillo *et al. *(2006) [26] presented two GO term examples having different contributions of data types. However, this information was used to explain the nature of the supporting genomic data types in the prediction results, but was not incorporated into the prediction methods. In other words, these examples are independent from the nine methods used in [26]. Our previous work in [26], one of the nine bioinformatics teams evaluating their methods for mouse function prediction, incorporated informative genomic data types into the KLR method for each GO term. The previous method, similar to the exhaustive search feature selection approach in this paper, has been among the best prediction groups in the general category of {31-100} and {101-300}, though it showed relatively poor prediction performance in the specific category of {3-10}. The KL1LR method introduced in this paper significantly improved the prediction quality in the category of {3-10}. As a result, the prediction performance of the KL1KR method was comparable to the best approaches in [26] using the same data sets, although the performance of the KL1KR method was still lower in the categories of {3-10}. It is difficult to directly compare the AUC values of these methods because the performance evaluation in this paper is based on the five-fold cross validation, whereas [26] is based on a held-out blind test set. However, the overall performance might be compared by considering that they are assessed in the same data sets. One of the best prediction groups in the categories of {3-10}, {11-30}, {31-100} and {101-300} of the biological process had respective AUC values of around *0.873*, *0.872*, *0.881*, and *0.84 *(Group C in Figure 2(a) of [26]) for the held-out blind test; those for the KL1LR method were *0.75*, *0.88*, *0.88*, and *0.86 *for the five-fold cross validation.

In the results section, we presented the prediction accuracy using the AUC value. In the Additional Files, various recall values are also calculated for each GO term and data source. It should be noted, however, that the AUC value might misinterpret the prediction when the number of the positive genes is smaller than the number of negative genes, though this can be compensated for by using the various recall values. Hence, we used the P20R threshold as one of the thresholds to select informative features, as shown through Tables 2, 3, and 4.

Furthermore, we analyzed the informative features of each GO term and the tendencies for informative genomic data types in the parent-offspring relationship in the GO term hierarchy. During the enrichment test in Table 4, we observed that the informative data sources for the GO terms are not independent of each other. As such, this observation can be used to infer the informative genomic data sets for the GO terms, even though their relationships with genomic data sets have not previously been analyzed. In an effort to guide the informative data sources for GO terms such that the use of this information by experimental and computational biologists who will collect data sources and predict protein functions can be made easier, a website [51] has been constructed. With the input of GO terms, the AUC values and various recall values based on the exhaustive search are listed. Using this website, biologists who plan for the prediction of functions of new GO terms are informed which data sources are needed to be collected first; users can also explore the parent's GO terms. This developement helps to deterimine the informative data sources even though the GO term has yet to be analyzed.

## Conclusions

For the assessment of contributions of genomic data sources for protein function prediction, we proposed the KLR method that uses the exhaustive search feature selection approach and the KL1LR method. The reason this protein function prediction is successful is due to the estimation of contributions of the data source, which directly influences the prediction performance. Our approach has the ability to find the relationship between protein functions and genomic data types. Moreover, our approach can be applied to any bioinformatics field that uses the high dimensional data sources. Therefore, it can be used as indispensable methodology for analyzing the subsequent genomic data.

## Authors' contributions

SK implemented methods of the feature selection, analyzed the data and drafted the manuscript. HL initiated and directed the research, developed integrative methods, and helped in writing the manuscript. All authors read and approved the final manuscript.

## Supplementary Material

Additional file 1 **Prediction results of each GO term**. The AUC values and regression model coefficients of KLR based on the exhaustive search feature selection and the KL1LR method related to Table 1 are represented. Information about the gene count of GO terms in each data source is also included since each data source has different coverage. Each data source was reduced to two features, as in Eq. (4), so, there are two features for each data source. Click here for file

Additional file 2 **Precision at various recall values**. Precision at various recall values for the ES and KL1LR methods (*λ *= *0.01*, standardized). Click here for file

Additional file 3 **Prediction result of each GO term using the Relief method**. The AUC values and regression model coefficients of the Relief method related to Table 1 are represented. Click here for file

Additional file 4 **The lists of GO terms predicted well based on a given data source in two different approaches**. To form these groups of GO terms, as high contribution criteria, high prediction accuracy (≥ 0.75 AUC and ≥ *0.2 *P20R value) and large coefficient (outside of *1*SD and ≥ *0.2 *P20R value) were used for the exhaustive search and KL1LR, respectively. Click here for file

Additional file 5 **GO terms giving high prediction quality from only one data source**. The table showing the entire lists of the data presented in Table 3. Click here for file

Additional file 6 **Underlying data of Table **3** and Additional File 5**. The table with the underlying data of Table 3 (Additional File 5). Click here for file

Additional file 7 **Underlying data of Figure **2. The table with the underlying data of Figure 2. Click here for file

Additional file 8 **Underlying data of Figure **3. The table with the underlying data of Figure 3. Click here for file

Additional file 9 **Underlying data of Figure **4. The table with the underlying data of Figure 4. Click here for file

Additional file 10 **Enrichment test result**. The entire lists of the data presented in Table 4. Click here for file

Additional file 11 **Hierarchy of 'Reproduction' (GO:0000003)**. The hierarchy of 'Reproduction' that has a high significant value based on MGI phenotype in the enrichment test is depicted. The dotted line describes an ancestor that is not a direct parent. In addition, 'Na' in parentheses indicates that a prediction cannot be achieved when the number of gene products in the MGI phenotype is not sufficient for cross validation. For example, in the hierarchy, the total number of gene products of a 'Viral infectious cycle' (GO:0019058) is four, but the MGI phenotype data source only has data about one of them. Click here for file

Additional file 12 **Prediction results of newly annotated mouse genes**. AUC values and regression model coefficients related to Table 5 are presented. Click here for file

Additional file 13 **Precision at various recall value of newly annotated genes of mouse**. Precision at various recall values for ES and KL1LR (*λ *= *0.01*, standardized). Click here for file

Additional file 14 **Prediction results of yeast genes**. AUC values and regression model coefficients related to Table 6 are presented. Click here for file

Additional file 15 **Precision at various recall value of yeast gene precision**. Precision at various recall values for ES and KL1LR (*λ *= *0.01*, standardized). Click here for file

Additional file 16 **Informative data sets table of newly annotated mouse genes**. Informative data sets from newly annotated mouse genes related to Table 7 are listed for each GO term. Click here for file

Additional file 17 **Informative data sets table of yeast genes**. Informative data sets from newly annotated yeast genes related to Table 7 are listed for each GO term. Click here for file
