# Supplementary figures and images for: Integrative approaches to the prediction of protein functions based on the feature selection
Source: BMC Bioinformatics. 2009 Dec 31;10:455. doi: 10.1186/1471-2105-10-455 (PMC2813249; doi:10.1186/1471-2105-10-455)

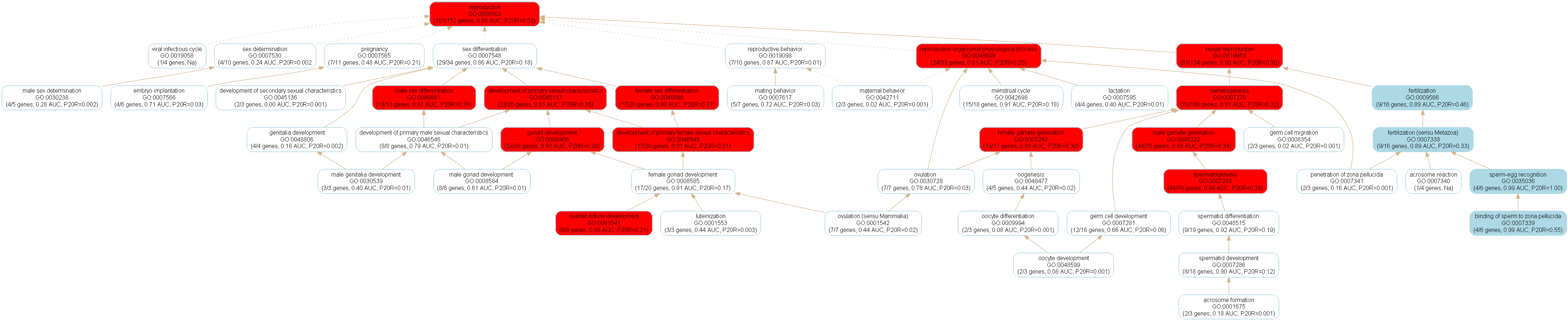

Supplement: Additional file 11 — Hierarchy of 'Reproduction' (GO:0000003). The hierarchy of 'Reproduction' that has a high significant value based on MGI phenotype in the enrichment test is depicted. The dotted line describes an ancestor that is not a direct parent. In addition, 'Na' in parentheses indicates that a prediction cannot be achieved when the number of gene products in the MGI phenotype is not sufficient for cross validation. For example, in the hierarchy, the total number of gene products of a 'Viral infectious cycle' (GO:0019058) is four, but the MGI phenotype data source only has data about one of them. [file 1471-2105-10-455-S11.PNG]
